# Analysis of the New Kuznets Relationship: Considering Emissions of Carbon, Methanol, and Nitrous Oxide Greenhouse Gases—Evidence from EU Countries

**DOI:** 10.3390/ijerph18062907

**Published:** 2021-03-12

**Authors:** Mara Madaleno, Victor Moutinho

**Affiliations:** 1 GOVCOPP—Research Unit in Governance, Competitiveness and Public Policy, Department of Economics, Management, Industrial Engineering and Tourism (DEGEIT), University of Aveiro, 3810-193 Aveiro, Portugal; 2 Management and Economics Department and NECE-UBI, University of Beira Interior, Rua Marquês d’Ávila e Bolama, 6201-001 Covilhã, Portugal; ferreira.moutinho@ubi.pt

**Keywords:** greenhouse gas emissions (GHG), gross domestic product per capita (GDPpc), Environmental Kuznets Curve (EKC), former European Union (EU 15) countries, new European Union (EU 12) countries, fully modified ordinary least squares (FMOLS), dynamic ordinary least squares (DOLS)

## Abstract

Decreased greenhouse gas emissions (GHG) are urgently needed in view of global health threat represented by climate change. The goal of this paper is to test the validity of the Environmental Kuznets Curve (EKC) hypothesis, considering less common measures of environmental burden. For that, four different estimations are done, one considering total GHG emissions, and three more taking into account, individually, the three main GHG gases—carbon dioxide (CO_2_), nitrous oxide (N_2_O), and methane gas (CH_4_)—considering the oldest and most recent economies adhering to the EU27 (the EU 15 (Old Europe) and the EU 12 (New Europe)) separately. Using panel dynamic fixed effects (DFE), dynamic ordinary least squares (DOLS), and fully modified ordinary least squares (FMOLS) techniques, we validate the existence of a U-shaped relationship for all emission proxies considered, and groups of countries in the short-run. Some evidence of this effect also exists in the long-run. However, we were only able to validate the EKC hypothesis for the short-run in EU 12 under DOLS and the short and long-run using FMOLS. Confirmed is the fact that results are sensitive to models and measures adopted. Externalization of problems globally takes a longer period for national policies to correct, turning global measures harder and local environmental proxies more suitable to deeply explore the EKC hypothesis.

## 1. Introduction

The Kyoto Protocol was the focal point to raise awareness of the need to mitigate greenhouse gas (GHG) emissions. At that time, developed countries committed to reducing GHG emissions by at least 5.2% during 2008–2012 compared to 1990 levels. This became known as the first commitment period [1]. According to [2], to meet the Kyoto Protocol targets, it would be necessary for rich countries to reduce fossil energy use and consumption (primarily responsible for GHG emissions) by 1%, and for rich and poor, the energy productivity (energy/labor) should be reduced by 4% to 5%. In these circumstances, if everyone reduced productivity to 2.5%, global energy consumption would be reduced from 1.1% to 0.65% annually. To achieve the objective of complying with the imposed obligations, the European Union (EU) set up a system for measuring GHG emissions and implementing an emissions trading system [3]. In the second commitment period—2013–2020—the countries that ratified the Kyoto agreement agreed to reduce these same emissions by 20% compared to 1990 levels. Even before this deadline, in 2015, the Paris Agreement was signed and 195 countries committed to keeping the global average temperature below 2 °C.

Consequently, in the EU, new environmental and new energy targets for 2030 were adopted. Among these measures, there is a required reduction of at least 40% in GHG emissions, as compared to 1990 [4,5] levels. Additionally, within Europe, several efforts have been made to reduce emissions, due to the strong environmental impact they represent, but also due to the serious consequences, they represent in economic and social terms [3]. To reinforce this commitment, the European Commission committed itself in Madrid in 2019, at the COP25 Climate Summit, to what became known as the European Green Deal. Thus, and by 2050, the EU should become climate neutral, but for that purpose, by 2030, CO_2_ emissions should be reduced to 50%, as compared to 1990 values [3].

In 2016, a directive was also approved to limit GHG emissions. Each of the European countries has well-defined targets for the years 2020 and 2029, as well as for subsequent years [6], after 2030. The main responsible for global warming are the greenhouse gases carbon dioxide (CO_2_), nitrous oxide (N_2_O), and methane (CH_4_). Other pollutants, like particulate matter, on the other hand, are responsible for the damage caused locally, precursors of tropospheric ozone, and for the particulate material emitted to the atmosphere [7]. Normally, gases are distinguished by their useful life, with CH_4_ with a lifespan of 12 years being considered a short-lived gas, and both CO_2_, which persists the atmosphere for hundreds of years, and N_2_O that persists for more than 100 years, being considered long-lived gases [8].

Both increases in pollution and climate change have been the driver for awakening societal interest in the relationships between economic growth and the environment. In a 2010 work [9] suggests that economies could follow balanced growth trajectories, while simultaneously cutting back on part of the pollution generated by economic activity. The so-called Environmental Kuznets Curve (EKC) reflects this relationship between economic growth and environmental quality, being not a permanent relationship, but variable depending on the phase of economic growth in which a country or region is at a given time. There are three possible explanations in the literature in support of the EKC. Firstly, the “relative variation in the values of marginal utilities of economic growth and environmental quality according to the growth of GDP per capita”. According to this, economies presenting lower income levels, have lower rates of return in reducing pollution, as compared to those due to increased consumption of goods, where marginal gains derived from this consumption decreases. Besides, the losses associated with pollution are increasing, which results in a negative marginal gain (causing an inversion of the marginal values until the pollution decreases). The second explanation relies on the “pollution haven” effect, which consists of the relocation of industries with higher levels of pollution from more developed economies (and therefore, with greater environmental regulation) to economies with a lower level of development, leading to what is called “environmental dumping”; The last explanation is related to “the dynamics of sectorial recomposition, but which is interdependent with the effects previously described”.

Finding evidence of the EKC reveals that GHG generation increases with increased GDP up to a certain GDP level, known as the turning point. However, thereafter, it decreases despite further economic growth. Therefore, adverse environmental impacts caused by more GHG emissions would decrease at elevated GDP levels. Although providing EKC evidence in GHG contributes to better understanding the relationship between economic-growth-GHG emissions, it does not diminish the urgent need to implement effective emission reduction schemes.

Considering the 2009–2018 period it is highlighted in [10] that for both OECD and non-OECD countries, the annual tendency is for the CH_4_ and N_2_O emissions to increase, whereas CO_2_ emissions are in a decreasing pattern. The latter, predominantly coming from the energy and industrial sectors, is the most dominant fraction of the total greenhouse gases, as referred in the Commission’s Report to the European Parliament, within the framework of the European Ecological Pact in 2019 [11]. There we can read that “*more than 75% of the EU’s greenhouse gas emissions result from the production and use of energy in all economic sectors*”. It is based on sectoral dynamics, in which the largest source of anthropogenic emissions is considered. The report points out N_2_O emissions due to agriculture, with this sectoral activity also having a predominant role in CH_4_ emissions [12], with CO_2_ emissions deriving mostly from the burning of fossil fuels.

A great part of the literature exploring the EKC hypothesis uses CO_2_ emissions to represent environmental degradation. Most of these studies confirm an inverted U-shaped relationship between economic growth and CO_2_ emissions [13]. However, the factors involved range from ecological footprints to GHG [14,15,16,17]. Using solely CO_2_ is very limited, provided environmental degradation and damage cannot be solely captured or analyzed through carbon emissions [18]. GHG emissions are used as proxies of environmental degradation (not validated in [19] for the EU 27, while for the EU 27 [20] found mixed results).

Recent studies provide in depth literature reviews in the research fields of air pollution and child health [21], on the impact of nanomaterials on the environment [22], and the environmental Kuznets curve research [23]. From these literature review examples, it is clear that countries’ environmental degradation runs in parallel with the economy and that health effects arise from here [24,25]. In [24] evidence for the relationship between particulate matter (PM) exposure and health effects (specifically, cardiopulmonary morbidity and mortality) is presented. Thus, linking economic growth to pollution emissions, from the EKC point of view, brings as well important insights into its public health implications. Therefore, a broader debate emerges besides the simple legally mandated air quality standards and imposed by EU legislation [21]. This link between the EKC hypothesis regarding economic growth and pollution effects is thus strengthened by the emergence of the consequent health impacts [21,22,23,24,25].

Additionally, [25] questioned the link between air pollution and the coronavirus disease 2019 spread (COVID-19), exploring the literature linking air quality (as measured by different pollutants) to health effects in general, and the new pandemic in particular. It is even argued in the literature that air quality provokes different impacts. On one hand, long-term exposure to particulate matter weakens health in general and lungs in particular. On the other hand, EU increased production and the documented negative effects caused by excessive gas emissions, will deeply affect population health, and the nature of air pollution should thus be accounted for in the growth-environment nexus, justifying the exploration of the EKC hypothesis considering different types of pollutant gases.

Income and social progress have been high in the European area [26], at least until the recent pandemic. The relatively high number of countries composing the EU 27 group, the environmental challenges, regulations imposition, the Sustainability Development Goals, and economic progress and environmental consequences imposed by this progress, justify further studies in this region. Moreover, under the EU legislation umbrella, governments of the EU 27 countries are forcing stricter national legislation to reduce emissions and pollutants, such as to achieve imposed targets. Furthermore, we motivate our study within the EU context since these are relatively energy-efficient and have several ongoing and past economic policies to address the environmental-economic growth nexus. They have implemented diverse policies and the EU 27 are very heterogeneous (diversity, different economic development stages, different environmental improvements), turning them a rich research opportunity and a motivation for analysis of the EKC hypothesis.

Thus, the main objective of this study is the simultaneous analysis of the validation of the Kuznets curve considering the total GHG emissions as a first estimation step, but also the EKC analysis by type of greenhouse gases, given that we admit, based on the literature review, that a trend of reduction or increase in emissions of greenhouse gases CO_2_ may not necessarily be accompanied by a similar change in CH_4_ or N_2_O emissions, so the effects of economic growth measures can influence these same emissions to different extents and in different directions if we consider solely GHG emissions in aggregate terms. Thus, in this study, we considered three different estimates for the analysis and validation of the EKC hypothesis with the three main greenhouse gases—CO_2_, N_2_O, and CH_4—_taking into account the oldest and most recent economies to accede to the EU 27, so we divide the EU countries into the EU 15 (old Europe) and the EU 12 (new Europe), and the results reveal a disparity between both groups. We have considered, for the validation of the EKC, as dependent variables, the total volume of GHG emissions per capita, and also its three main components, namely, the volume of emissions of carbon per capita, emissions of methanol per capita, and emissions of nitrous oxide per capita. As explanatory variables, we considered the value of the gross domestic product per capita (GDPpc), and some exogenous possibly influencing variables, also commonly employed in the relevant literature, such as labor force, energy use, and electricity production. We have considered all variables weighted by the geographical area of a given European country in the sample, which is a is another novel contribution to the existing literature.

This approach, which aims to show EKC’s analysis taking into account the diversity of European Union countries in economic, demographic, and geographic terms (see [27]), turns EKC’s piecemeal analysis by type of greenhouse gases into a research opportunity, having an important gap in the analysis of the EKC relationship been detected in the reviewed literature, which is presented in the next section. Furthermore, our results make evident the mixed findings and stages of the countries regarding the EKC hypothesis validity provided it was only possible to be validated in the EU 12 country group, under a specific methodological condition, turning results sensitive to the years of entrance in the EU community, methodology, and type of polluting gases. This in turn highlights important policy measures to be undertaken which have been explored in depth in the policy implications section.

## 2. Framework and Literature Review

Through time, both theoretically and empirically, the literature has explored the GDPpc impact on environmental degradation. Positive effects were usually found in the short-run imposing higher growth associated with more pollution. However, in the long-run, it has been stated that whenever GDPpc grows up to a certain level, after crossing this turning point, environmental degradation starts decreasing, even with continued growth. This phenomenon became known in the literature as the Environmental Kuznets Curve (EKC) hypothesis and has been studied in depth ever since [13,28,29,30,31,32,33].

Kuznets presented the inverted U-shaped relationship between economic growth and income inequality. These dimensions would present a positive relationship up to the turning point, after which, an increase in economic growth causes increases in income inequalities [34]. The literature is consensual at attributing to [35] the implementation of the Kuznets curve in environmental economics. This economic growth has a relation with pollution levels, where, once again, they have a positive relationship up to a certain point (which varies country by country) where the relationship is reversed [20].

The logic behind the EKC hypothesis is that at the initial phase pollution increases due to the strong industrialization given that the priority is economic growth, production, and expansion [36,37,38]. During this phase, attention is given to income at the cost of decreased attention to clean air and water. Moreover, technology is intensively used to exploit resources, leading to lower financial resources to pay for abatement. As well, regulation is scarce and natural resources are overused, leading to environmental degradation with all the associated costs (wealth, wellbeing, unsustainability, etc.). In the second stage or the long run, as countries switch from developing to developed economies, and as science, research and technology evolve, greater attention is paid to the environment. At this stage, stricter environmental regulations are implemented, increasing awareness and discouraging further environmental damage due to production and consumption practices. With increased economic security, attention is focused on the reduction of pollutant emissions and concentrations [13,15].

The wider measures used as a proxy for environmental degradation include GHG emissions. Mostly used are narrow ones like sulphur and carbon. CO_2_ is the most used, but we also find studies applying CH_4_ [39], N_2_O [39,40], nitrogen dioxide (NO_2_) [41], nitrogen oxides (NOx) [42], sulphur dioxide (SO_2_) [43], non-methane volatile organic compounds (NMVOCs) [44], sulphur oxides (SOx) [43], particulate matter with a diameter of 10 µm or less (PM_10_) [41], ecological footprint [18,45,46] and coal consumption [47]. Reference [48] points out that the EKC hypothesis and the empirical literature are sensitive to the choice of the environmental burden measure adopted, with the validation of the hypothesis when local pollutants are used, not being true when the measures adopted are global emissions (like CO_2_). Several authors have tested this relationship since the 1990s, using different variables (GHG emissions, polluting gases, ecological, water, and waste indicators, etc.) as environmental indicators [20]. Reference [19] highlights that there is a very large focus on the Kuznets environmental curve, which relates the product to CO_2_ emissions. Reference [49] explained that the applicability of the Kuznets environmental curve has been demonstrated for pollutants such as SO_2_, but not for greenhouse gases, even giving an example in which an effective turning point [50] of $8 million per capita was found, as high as it is unrealistic. Even so, there are still arguments to support the validity of the Kuznets environmental curve even for this type of emissions, and the model continues to have adherents, even though [13] consider the estimates of this curve to be inconclusive for CO_2_, the gas responsible for the highest emissions of greenhouse gases,. One of the authors that [19] mentions, having also studied the relationship between the three dimensions, more specifically in France, was [51], who concluded that growth leads to a long-term increase in emissions of CO_2_ and energy consumption.

Using e-waste as an example, [26] tested the validity of the EKC in the EU28+2 during the 2000–2016 period using the generalized-method-of moments (GMM) estimator, the two stages least squares (2SLS) estimator, and the cross-section method. They support the validity of the EKC for e-waste management arguing that the relationship is robust and not sensitive to the choice of control variables nor estimation methods. Results from the study highlight that the turning point happens at very high GDP levels. Reference [17] used two indicators of environmental degradation (suspended particulate matter (SPM) and SO_2_) applying OLS. For the 33 countries analyzed during 1979–1990, they found evidence of the EKC hypothesis validity but just when using SPM and GDPpc. Taking into account the differences in the economic structure of the seven European countries analyzed and not overall growth as usual, [52] explored the existence of an inverted U-shaped relationship. They apply empirically the stochastic (ST) estimation of environmental impacts (I) by regression (R) on population (P), affluence (A), and technology (T) (STIRPAT) model and fully modified ordinary least squares (FMOLS) estimation techniques. They also use CO_2_ emissions as a representative of environmental degradation but the added value of an industry as a proxy for economic growth. They explored data solely during 1980 and 2014. Reference [53] confirmed the validity of the EKC hypothesis for the EU 28 panel using SOx and NMVOCs data during 1990–2014. They included other types of emissions like CO_2_, NOx, and NH_3_ and also applied pooled estimations, fixed effects, and panel vector error correction models. They confirmed the neutrality hypothesis verifying the causal link between economic growth and primary energy consumption. Reference [46] also empirically analyzed the EKC hypothesis considering as the environmental degradation variable the ecological footprint. On the right-hand side of their equation, the authors included economic growth, energy consumption, and population growth. Pooled mean group and augmented group models were used to estimate long-run parameters for 22 EU countries considering the 1995–2015 period. The FMOLS and dynamic ordinary least squares (DOLS) techniques were employed for robustness checks, to help advise the adoption of policies able to restrict emissions, deforestation, air, land, and water pollution, if the goal in EU is to ensure environmental sustainability. Population growth was found not to influence the environmental quality, suggesting the possibility of adoption of greener and more advanced technologies in the EU countries (as stated by [23]).

Also [54] studied the relationship between energy consumption and CO_2_ emissions, specifying some sectors in Taiwan that should reduce their energy intensity, given the predominant impact of energy production and use CO_2_ emissions, by far, the gas most representative of greenhouse gases. Reference [54] utilized carbon dioxide emissions and ecological footprints as proxies for environmental degradation, during 1990–2014 for 14 countries. The results suggest the presence of an inverted U-shaped curve, and studying the causality, the authors found a feedback relationship between ecological footprint and renewable energy sources, and a unidirectional link from economic growth to environment degradation. Still in Europe, [55,56] investigated the EKC hypothesis adding the biomass consumption to the model, for 24 countries (1980–2010) with an autoregressive distributed lags (ARDL) model procedure. The results indicated the presence of the EKC proposition and concluded for biomass consumption is negatively related to CO_2_ emissions. Reference [57] analyzed the EKC relationship (environmental degradation and economic growth) and energy innovation for 33 European countries through FMOLS. The results support the Kuznets curve hypothesis and the authors argue how provide answers to policymakers, governments, individuals, and businessmen on how to increase the growth of business and economy without harming the environment. Reference [18] used a broader proxy, namely ecological footprint, to study the EKC hypothesis for only 15 EU countries and during 1980–2013, confirming a U-shaped relationship. Furthermore, it is stated that non-renewable energy increases environmental degradation, whereas renewable energy and trade openness decrease it. However, different countries gave mixed findings for applying two different methodologies (FMOLS and DOLS) drives different results.

As inferred from the above, the studies differed in the methodological terms used to validate the Kuznets curve, depending on the data structure used, through time series [58,59,60,61] or panel data [18,20,62,63]. The analysis of the relationship between economic growth and GHG emissions started with a study carried out by the World Bank. This study with panel data from 1980 to 1990, found significant evidence that proved a relationship with increasing trends between economic growth and CO_2_ emissions [64]. Reference [65], studied for the EU-12 group the relationship between GDPpc and SO_2_ emissions, from 1870 to 2001. To do so, they adopted the fixed and random effects model, concluding the existence of the Kuznets environmental curve. Regarding the EU-15, [66], using the ARDL methodology, studied the relationship between GDPpc and the waste indicator for 1997 to 2001, finding no evidence of the Kuznets environmental curve. The same conclusion was verified by [18] for 1980 to 2013, with the application of ARDL models with panel data, such as mean group (MG), FMOLS, and DOLS to study the relationship between ecological footprint, GDP, trade openness, energy consumption and consumption of renewable and non-renewable energy. Also for the EU-15, [67], using DOLS, and [61], using OLS, found significant evidence to prove the existence of the Kuznets Curve. Reference [20], a study for the EU 27 (1995–2010), found mixed results for the relationship between GHG and real GDP (the justification given for the presence of mixed results concerns the presence of several economic factors, environmental policies, and the income level).

Before we move on and since the literature relies greatly on FMOLS and DOLS methodologies, we find it relevant to explain briefly what these models represent in this context. The DOLS estimator addresses the problems of endogeneity and autocorrelation of errors through two corrections. The first, to deal with the problem of endogeneity, tries to apply a linear projection of current errors (*z*_t_*) on past, contemporary, and future errors to eliminate the contemporary correlation between regressors (*y*_2*t*_) and errors (*z*_t_*). As such, an equation is then increased by the lags and leads to the new errors constructed from the linear projection of *z*_t_*. The second correction serves to face a possible problem of the autocorrelation of the errors of the linear projection that may persist. This correction involves using the Newey-West estimator [68]. Note that, once we start using a dynamic model (due to the use of leads and lags in the linear projection of errors *z*_t_*), the problems of autocorrelation may disappear. Another estimator to be used in this work is FMOLS developed by [69]. Through the OLS estimation, this estimator tries to make semi-parametric corrections in two steps to face the problems of error autocorrelation (based on [70] in the scope of the unit root test) and endogeneity of the estimator regressors OLS statistic. For a brief and deeper explanation of the procedure, see [71]. Again, as with DOLS, we have the advantage with this estimation method over OLS of making inference possible. Since the FMOLS has the same asymptotic behaviors as the maximum likelihood methods, the t and Wald tests have the usual asymptotic normal and chi-square distributions, respectively.

With an FMOLS approach, [72] studied 22 members of OECD, during 1971–2000 and found support for the EKC hypothesis, a similar result was found by [73,74]), and besides the research described in [73] tested for causality. The results point to evidence of a two-dimensional relationship between emissions and GDP, investigating 78 countries, 26 OECD members with high income and 52 developing economies, applying an OLS panel estimation for the period of 1980–2010, but they do not support the EKC hypothesis for any of the countries in the sample [75], while [76] only supports the EKC for the short term, with a pooled OLS (POLS) and DOLS approach for 36 OECD countries during 2000–2017.

From the above presentation, our contributions are clear. As different pollutant gases exert different effects on the environment, the measure of economic growth can influence these same emissions differently (in magnitude and direction). Thus considering only overall GHG has limits. To test the EKC hypothesis, besides global GHG emissions, we use the three main greenhouse gases (CO_2_, N_2_O, and CH_4_), as dependent variables. Furthermore, and given the different commitments faced by countries in the EU, we took into account the oldest and most recent accessions of economies to the EU 27 (EU 15 (“old Europe”) and the EU 12 (“new Europe”)). Results reveal the disparity between both groups in terms of the EKC validation. As explanatory variables in the EKC equation, we considered the value of the gross domestic product per capita (GDPpc) and its squared value, and commonly employed independent variables by the literature, such as labor force, energy use, and electricity production. However, none of the previous works considered all these variables weighted by the geographical area of the respective European country in the sample, being this an additional contribution and a novelty of this work. This is done because all EU countries are heterogenous and the geography covered is related to pollutant gas emissions [27]. Therefore, our goal is to test the validity of the EKC hypothesis taking into account the diversity of European Union countries in economic, demographic, and geographic terms (see [27]). In this respect, previous research turns EKC’s piecemeal analysis by type of greenhouse gases into a research opportunity, having an important gap been detected in the reviewed literature on the analysis of the EKC relationship. Results presented in the following sections highlight the differences among EU groups which depend on the specific methodology employed, turning results sensitive to the years of entrance in the EU community, to the methodology used, and the type of polluting gases considered. Different policy implications emerge from these scenarios.

## 3. Data and Methodology

### 3.1. Data and Selected Variables

We try to analyze and validate the EKC hypothesis, which focuses essentially on the relationship between GHG emissions and economic growth. Three different GHG emissions have been as well considered for each group of countries, to infer differences in terms of validation of the curve. This study is carried out with a sample panel data for 27 countries belonging to the European Union in the period from 2008 to 2018. Two subsamples were selected to ascertain whether there are differences regarding the validation of the EKC relationship in the “old Europe” (EU-15) and the “new Europe” (EU-12). Further explanatory variables able to influence the relationship and commonly reported in the literature as influencing the EKC relationship (labor force, energy use, electricity production), are described in Table 1, where we summarize information about the variables considered and the sources of access to statistical information.

### 3.2. Methodology: Cointegration in Panel Data

In the analysis and validation of the Kuznets environmental curve, we propose the estimation of the short and long-term relationship, considering two important aspects. The first was based on the work developed by [19], in which this author, when studying the effects of energy on emissions GHG, considered two variables inserted in the right-hand side of the equation such as energy use (kg of oil equivalent pc) and per area, and electricity production from oil, gas and coal sources (% of total) per area. The second aspect was supported by the work of [27] when analyzing the GHGs by homogeneous groups concerning GDP, GDP pc, and surface area in km^2^. The percentage of the working-age population with an advanced level of education per capita and the per area has been included to represent the workforce and production factors available in the economy, as well as the capacity to deal with newer technologies, innovation, and with hope to capture human capital effects. In the present work, we propose the following four equations to explore the EKC hypothesis:

(1) $$\frac{GHG_{pc}}{Area}=\beta_{1}+\beta_{2}\;\frac{GDP_{pc}}{Area}+\beta_{3}\frac{GDPpc^{2}}{Area}\;+\beta_{4}\;\frac{LEdu_{pc}}{Area}+\beta_{5}\;\frac{Euse_{pc}}{Area}+\beta_{6}\;\frac{Elect_{pc}}{Area}+µ$$

(2) $$\frac{CO2_{pc}}{Area}=\beta_{1}+\beta_{2}\;\frac{GDP_{pc}}{Area}+\beta_{3}\frac{GDPpc^{2}}{Area}\;+\beta_{4}\;\frac{LEdu_{pc}}{Area}+\beta_{5}\;\frac{Euse_{pc}}{Area}+\beta_{6}\;\frac{Elect_{pc}}{Area}+µ$$

(3) $$\frac{CH4_{pc}}{Area}=\beta_{1}+\beta_{2}\;\frac{GDP_{pc}}{Area}+\beta_{3}\frac{GDPpc^{2}}{Area}\;+\beta_{4}\;\frac{LEdu_{pc}}{Area}+\beta_{5}\;\frac{Euse_{pc}}{Area}+\beta_{6}\;\frac{Elect_{pc}}{Area}+µ$$

(4) $$\frac{N2O_{pc}}{Area}=\beta_{1}+\beta_{2}\;\frac{GDP_{pc}}{Area}+\beta_{3}\frac{GDPpc^{2}}{Area}\;+\beta_{4}\;\frac{LEdu_{pc}}{Area}+\beta_{5}\;\frac{Euse_{pc}}{Area}+\beta_{6}\;\frac{Elect_{pc}}{Area}+µ$$

where the variables have been described in Table 1. Equations (1)–(4) have been estimated separately for the EU 27, the EU 15, and the EU 12.

To validate these four equations we considered short and long-term relationships between the variables included in the four equations. The panel data sets (one for EU 27, another for EU 15, and another for EU 12) are estimated using the pooled mean group (PMG) estimator described by [77,78], and the mean group (MG), and dynamic fixed effects (DFE) proposed by [79]. We have started the empirical analysis pursuing the application of diagnostic tests. Subsequently, a panel unit root test was applied to verify if the variables are indeed non-stationary. Only after guaranteeing the non-stationarity of variables and cointegration among them, may we analyze which variables’ deviations from the long-run equilibrium are influencing the short-run dynamics of the model. The co-integration vector was determined afterwards.

The PMG estimator as well as the MG estimator allow the intercepts, the short-term coefficients, and the error term to vary between groups, whereas the PMG estimator also restricts the long-term coefficients to be the same across the panel. In the DFE estimator all parameters are assumed to be homogeneous for all panel countries, except the intercept time (varies between cross-sections). For our sample of countries, the long-term equilibrium relations between the explanatory variables and the dependent variable for the two EKC specifications seem to be similar (the countries are subject to the same conditions as energy policies, GHG emissions mitigation policies, economic cycles, among others). Despite the fact we used specifications for all three estimators, the PMG method is expected to be preferable to the MG and DFE. Considering the possible endogeneity and serial correlations of regressors, the FMOLS estimator, recommended by [80], will be used, as well as the dynamic ordinary least squares (DOLS), suggested by [81]. They differ in the way the observations are combined. Following [80], group-means estimators should have greater flexibility over the existence of heteroscedasticity in the co-integration vectors, but pose a better size distortion, leading us to use these versions for the FMOLS and DOLS estimators.

#### 3.2.1. Diagnostic Tests

Cross-section dependence is tested following [80], where it is proposed an alternative general cross-sectional dependence test (CD):

(5) $$D=\sqrt{\frac{2T}{N\left(N-1\right)}}\;\left(\overset{N}{\underset{N-1}{\sum }}\overset{N}{\underset{j=i+1}{\sum }}\hat{\rho_{ij}}\right)$$

Under the null hypothesis of no cross-sectional dependence (CD), we will have that *D*→*N* (0, 1) for *N*→∞ and *T* sufficiently large [82]. Although the CD test is robust for heterogeneous dynamic models, with multiple breaks in the slope coefficients and error variations, its drawback is its lack of power under some circumstances where the sample average pairs of correlations are zero [55]. To validate results, we resort to [83] which is not subject to this problem, where the statistic is calculated over the sum of the square rank correlation coefficients and equals Equation (6) [83]:

(6) $$R_{ave}^{2}=\;\frac{2}{N\left(N-1\right)}\overset{N-1}{\underset{i=1}{\sum }}\overset{N}{\underset{j=i+1}{\sum }}{\hat{r}}_{ij}^{2}$$

#### 3.2.2. Unit Root Tests

To check data stationarity we have employed four different tests. The Im, Pesaran and Schin (IPS), Levin, Lin and Chun (LLC), Maddala and Wu (Fischer PPerron), and the Hadri tests. The first (IPS), specifies an ADF-type regression for each cross-section unit and then calculates the mean of the type t statistics for the $\beta_{i}$ coefficient to reach the panel test statistic [84]. The second (LLC) test uses a homogeneity alternative hypothesis, which derives coefficient estimates from proxies of ∆y_it_ and y_it_. The LLC test does auxiliary regressions on lagged values and exogenous variables [84]. The third (Maddala and Wu) allows the application of individual regressions for each panel unity using a Phillips-Perron (PP) specification. It combines the p-values found in the individual test for a unit root in each of the panel’s cross-sections [85]. Finally, the Hadri Lagrange multiplier (LM) test has as the null hypothesis that all the panels are (trend) stationary, allowing to include fixed effects and time trends in the model of the data-generating process.

#### 3.2.3. Estimation Methodology

To validate the EKC hypothesis, the current literature uses a traditional specification from ARDL (p,q_i_):

(7) $$y_{it}=\;\sum_{j=1}^{p}\alpha_{ij}y_{i,j-t}+\sum_{j=0}^{p}\beta_{ij}X_{i,t-j}+\mu_{i}+\varepsilon_{it}$$

being *p* the number of lags of the dependent variable, *q* the number of lags from the independent variables, where *i* = 1, 2, …, *N*, *t* = 1, 2, …, *T*. *X_it_* represents the vector (*k* × 1) of independent variables, and $\beta_{ij}$ the vector of unknown parameters. $\alpha_{ij}$ are scalars, μ_i_ is the specific term from each country and $\varepsilon_{it}$ stands for the error term [84]. This approach is more suitable for the present study considering the number of countries in the sample. Moreover, if the series is stationary and the variables cointegrated, we can further consider that deviations from the long-term balance and influence the short-term [84]. This deviation answer is easily represented by an error correlation model (ECM):

(8) $$\Delta y_{it}=\;\emptyset_{i}\left(y_{i,t-1}-{\theta^{\prime }}_{i}X_{it}\right)+\sum_{j=1}^{p-1}\alpha_{ij}^{*}\Delta y_{i,t-j}+\sum_{j=0}^{q-1}\beta \prime_{ij}^{*}\Delta X_{i,t-j}+\mu_{i}+\varepsilon_{it}$$

where $\emptyset_{i}=\;-\left(1-\sum_{j=1}^{p}\alpha_{ij}\right)$, $\theta_{i}=\sum_{j=0}^{p}\beta_{ij}/\left(1-\sum_{k}\alpha_{ik}\right)$, $\alpha_{ij}^{*}=\;-\sum_{m=j+1}^{q}\alpha_{im}$ with j=1, 2, …, p−1, and $\beta_{ij}^{\prime *}=\;\sum_{m=j+1}^{q}\beta_{im}$ with j=1, 2, …, q−1.

To perform the estimations, we resorted to the PMG, MG, and DFE techniques. Developed by [79], the MG permits intercepts, coefficients, and errors to vary between groups in the short and long run. Therefore, panel estimations are derived from the arithmetic mean of the coefficients, individually computed for each cross-section [84], assuming the autoregressive distributed lag (ARDL) methodology form [79]. However, it makes estimations sensitive to shocks and outliers. As such, the PMG becomes an intermediate and alternative methodology, similar to MG in the short-run, but preventing the coefficients to change in the long-run (they are the same across panels). This has behind a maximum likelihood method that turns consistent and asymptotically normal the estimated coefficients whether I (1) or I (0), applying Equation (9):

(9) $$\hat{\theta }=-{\left\{\overset{N}{\underset{i=1}{\sum }}\frac{{\hat{\emptyset }}_{i}^{2}}{{\hat{\sigma }}_{i}^{2}}X{’}_{I}H_{i}X_{i}\right\}}^{-1}\left\{\overset{N}{\underset{i=1}{\sum }}\frac{{\hat{\emptyset }}_{i}^{2}}{{\hat{\sigma }}_{i}^{2}}X{’}_{I}H_{i}\left(\Delta y_{i}-{\hat{\emptyset }}_{i}y_{i-1}\right)\right\}$$

Similar in co-integration to the PMG estimator is the DFE estimator, which has to be homogeneous across all panels in the long run. It limits the adjustment coefficient speed and the short-run coefficient to be homogeneous [56]. We need to be aware that this technique might produce inconsistent results unless all coefficients are identical [55,84]. For the methodology applied to work, we need to ensure non-stationarity and cointegration of and amongst variables. Only then it is possible to conclude which variables’ long-run deviations from the equilibrium will impact the short-run dynamics. Our focus relies upon the parameters $\phi_{i}$ and $\theta_{i}$, namely, the adjustment speed from the error correction term and the long-run equilibrium relationship parameter vector. The former (term) is expected to differ from zero, while the latter (parameter) is supposed to be substantially negative. This under the premise that the variables return to their long-run equilibrium.

## 4. Empirical Results

Results presented in Table 2 support that all variables have cross-section dependence (H0 is rejected at the significance level of 1%), considering the global panel of EU 27. In turn, when considering both groups of EU 15 and EU 12 countries, individually, the results of the Pesaran CD Test point to the rejection of the null hypothesis for all variables, except the variable labor force with higher education (Edu pc/Area) for Europe 12.

Despite our previous results, in Table 3 we present the results of panel unit root tests. In the first-generation unit root tests, we include the Maddala and Wu test, while the Pesaran CIPS test was applied for the second-generation. Both have a purpose to verify the presence of unit roots for variables in levels and first differences.

Results of the first-generation unit root tests, both in levels and with the trend, for both EKC Equation (3) and EKC Equation (4), for the EU 27 and EU 12 panel countries, show that most variables are statistically significant at the 1% level or the 5% level, leading us to reject the null hypothesis, except for variables labor force with an advanced level of education, and electricity production from fossil fuels/surface area. For EU 15 countries, the statistical evidence of unit roots is presented for Equations (2)–(4), and all variables, except for labor force with an advanced level of education, meaning that most variables are integrated of order I (0).

The first differences results for the first-generation models, both in levels and without trend, show for the aggregate panel with the EU 27 countries and Equations (3) and (4), that most variables show statistical significance at 1% level or 5% level, except labor force with an advanced level of education and energy use (kg of oil equivalents per capita/surface area). In the EU 12, the results present for all four equations proposed and for all variables, statistical evidence to reject the null hypothesis. However, for the EU 15, both without and with the trend, for Equations (3) and (4), the results demonstrate statistical significance only for GDP per capita and GDP Quadratic per capita, turning most variables integrated of order I (1). As for second-generation unit root test results of Pesaran, both in levels and without trend, for all three panels, EU 27, EU 15, and EU 12, evidence for Equations (1) and (4), statistical significance to reject the null hypothesis in case of the variables energy use and electricity production from fossil fuels. However, both in levels and with the trend, the results for EU 27 and EU 15 countries, only show for Equation (4), statistical significance to reject the null hypothesis, that is, most variables are integrated of order I (0).

However, in first differences and without trend, for the Pesaran test, the results for the panel of the EU 27 countries, shows for Equations (2) and (4), statistical significance evidence to reject the null hypothesis for all variables, except, once more, for the variable labor force with advanced education. Statistical significance occurs for all four equations proposed, in the case of the panel of the EU 12 countries. Moreover, for the panel of the EU 15 countries, all variables selected in Equations (2) and (3) present statistical significance to reject the null hypothesis.

If we consider first differences with the trend, the results show for the panel of the EU 27 statistical significance to reject the null hypothesis only in two variables, namely, energy use per Kg of oil equivalent, per capita/surface area, and electricity production from fossil fuels/per capita/surface area. For the EU 12 panel those same variables show statistical significance for Equations (3) and (4), that is, most variables are integrated of order I (1). Moreover, the results of the CIPS test for the presence of dependence between cross-sections confirm some results showed by the first-generation of a unit root. Therefore, the results reinforce the conclusions regarding the presence of unit roots in most of the series of variables considered in each equation of the EKC proposed. In general, the assumption of non-stationarity of the series is legitimate, evidencing as well the possibility of admitting the existence of long-run relationships between variables.

Moving one step forward, we also tested for the existence of cointegration. Following the Table 4 results, cointegration test results are presented and further discussed and analyzed in the following. Both Pedroni and Kao tests of cointegration were performed. Under these considerations, the presence of cointegration supports the necessary condition for the balance between variables in the long run to exist. Pedroni’s test statistic, for all four questions proposed, reject the null hypothesis that there is cointegration at either the level of 1% or at the level of 5%, for the aggregate panel of the EU 27 and for the two samples of EU 15 and EU 12 countries selected. In turn, the Kao test statistics for Equations (1) and (2) do not reject the null hypothesis that there is no cointegration into EU 27, EU 15, and EU 12, either at 1% or 5%. It is also statistically significant leading to the rejection of the null hypothesis for the proposed Equation (4), for nitrous oxide per capita and per area, while leading to the non-rejection of the null hypothesis in Equation (3), when the EKC hypothesis is tested considering the emissions of methanol, this time at the 5% level of significance.

To analyze and to evaluate the EKC relationship, considering the four different measures of environmental degradation proposed, and to perform tests to verify the presence of these relationships in the four equations, use is made of estimation methods selected according to the limitations described in the analysis of the cointegration tests. Namely, the use of the PMG, MG, and DFE methods, which involve very restrictive hypotheses about the heterogeneity/homogeneity of the parameters are presented next. We also included the DOLS and FMOLS methods, as many of the previously analyzed authors did, that differ from the DFE because they perform the correction of the variables’ endogeneity.

With the performance of the Hausman test, it was possible to conclude either concerning the total sample Europe 27 countries, or about the subsample of EU 15 and the subsample EU 12. For the four equations there was a rejection of the null hypothesis, that is to say, the unobservable individual effects are not correlated with the model’s explanatory variables. According to the results obtained, it appears that the most appropriate model is the FE. The prevalence of a homogeneous panel indicates that countries share the same coefficients, which may be appropriate by treating them as a group in the EU. The results of Table 5, Table 6, Table 7 and Table 8, present the short and long-run elasticities/impacts for each of the four equations proposed for the study of the Kuznets relationship. In the short-run, they are represented by the coefficients of the first variables in differences, while in the long term they are based on the estimated coefficients of the respective lagged independent variables, divided by the lag of the dependent variable, multiplied by a negative sign.

The results of the DFE estimator show in the EKC relationship for the short term the U-shaped curve for Equations (1), (2) and (4) for panels EU 27, EU 15, and EU 12, while Equation (3) is only validated for panel EU 27 and EU 12 according to the expected signals and the estimated coefficients. Regarding the long-term validity of the EKC in a U-shape form, it only occurs in the estimation of Equation (1) and the estimation of Equation (2) for the panel of EU 15 countries. Thus, we can conclude based on this statistical evidence, that the high levels of greenhouse gas emissions are associated with high levels of economic growth, both at the aggregate level and the disaggregated level by two individual groups of countries, namely the old Europe EU 15 and the new EU 12, not validating the shape of the inverted U expected to be obtained to validate the EKC curve hypothesis, the so desirable effect.

Going deeper into the results for general GHG emissions, presented in Table 5, it is verified the U-shaped form for each of the countries group of panels only under the DFE specification. Only for EU 12 in the short run can we also validate the U-shaped relationship between GHG emissions and GDPpc in the short-run. Moreover in the short-run, LEdu is only non-significant and positive under the DFE specification for EU 27 and EU 12. In the long-run, the U-shaped form is only validated in DFE for EU 15. Euse and Elect are always significant and positive over GHG emissions in the short-run, indicating the negative contribution to increased emissions, turning harder the environmental burden in the EU. The rest of the variables in the long-run are not always significant and under some specifications, they have a contrary sign to the one expected.

Overall (Table 5, Table 6, Table 7 and Table 8), regarding the effect of the other variables considered in the first two proposed EKCs (Equations (1) and (2)), the results of the DFE estimation, both in the short-run and in the long-run, present statistical significance for the variables energy use Kg of oil equivalent per capita/surface area and share of electricity production from fossil fuels/surface area, in all three groups (EU 27, EU 15 and EU12) for Equation (1) and groups EU 27 and EU 12 in the case of Equation (2). The variable labor force with an advanced level of education shows in the short run significance in the EU 15 countries group and in the long-run, it shows significance for all three groups considered in the analysis, although with a sign contrary to what was theoretically expected. In Equation (3), the DFE estimator shows statistical significance in the variable energy use kg of oil equivalent per capita/surface area and share of electricity production from fossil fuels/surface area in the short-term estimates in the EU 27 and EU 12 groups. In the long-run, the results show only statistical significance for the variable labor force with an advanced level of education and only in the EU 15 group. In turn, in Equation (4), for the EU 27 and EU 12 groups, there is only statistical significance for the estimates in the short-run obtained for the coefficient of the variable energy use Kg of oil equivalent per capita/surface area.

However, in the long-run, the results show significant statistical evidence in all three groups of European countries considered only for the variable labor force with an advanced level of education. Besides, and not least, the error correction term (ECM) is highly significant in statistical terms, this value represents the speed of adjustment of the variables in the long-run equilibrium, which is fundamental for the understanding of the nexus between economic growth and greenhouse gases both in aggregate terms (Equation (1)) and in disaggregated terms (Equations (2)–(4)), we can say that the annual adjustment speed is slow, analyzing the FE estimators for panels EU 27, EU 15 and EU 12.

Accordingly, to the results shown in Table 5, Table 6, Table 7 and Table 8, the results of the DOLS and FMOLS estimation show the U-shaped curve for Equation (1) with statistical significance in the short-run and long-run relationship, respectively. The results of the DOLS and FMOLS estimation and for the three groups of EU countries, concerning the coefficients, are statistically significant in the short-run associated with the variables energy use Kg of oil equivalent per capita/surface area and share of electricity production from fossil fuels/surface area, while in the long-run it is necessary to record its importance with the FMOLS estimation and for EU 15, in the first proposed EKC relationship. In the DOLS and FMOLS estimation results, the statistical significance of the variable labor force with an advanced level of education for the three groups of European countries considered in the analysis is noted.

However, it should be mentioned that the results of the DOLS and FMOLS estimators, for the Equation (3) (Table 7) and Equation (4) (Table 8) proposed in our analysis, show sufficient statistical evidence to validate the inverted U relationship, only for the New Europe EU 12 economies. In the 3rd equation, the short-run and long-run coefficients associated with the GDP pc/surface area and GDP quadratic pc/surface area variables are positive and negative, respectively, with the application of the DOLS (short-run) and FMOLS (long-run) estimators. In estimating Equation (4), both in the short and long term, this same statistical evidence on the validity of the inverted U-shaped EKC only occurs with the application of the FMOLS estimator and for the same group from the New European countries.

Digging deeper into the results presented in the last four tables (Table 5, Table 6, Table 7 and Table 8), the U-shaped relationship between environmental burden and economic growth is confirmed under the DFE specification using overall GHG emissions for all European countries groups considered. Besides it is as well verified for the EU 12 group under the DOLS specification (Table 5), this for the short-run. In the long-run, this U-shaped relationship is also verified but only for the DFE specification and the EU 15. The same happens using CO_2_ emissions as a representative for environmental degradation, under the same model specifications and for the short-run. However, the U-shaped relationship is only verified in the long-run as well under the DFE specification, this time for the EU 12 (Table 6). Interestingly, when we use the more local emissions measurements like CH_4_, in the short-run, the inverted U-shaped relationship is evidenced under the DOLS technique but only for EU 12, the newly or most recent EU countries. Still, a U-shaped relationship emerges in the short-run for EU 27 and EU 12 using DFE, and under the FMOLS specification this happens in the short-run for the EU 27, and in the long-run solely under the DOLS specification for the EU 12 group (see Table 7).

Finally, using N_2_O as a representative of emissions, Table 8 shows evidence that in the short-run, the U-shaped relationship is still confirmed for the EU 27 and EU 15 groups under the DFE specification. It also happens for EU 15 using FMOLS, whereas in the long-run this same U-shaped form of the EKC is verified only under the DFE specification for EU 15 and EU 12. Curiously, the inverted U-shaped relationship desired is confirmed for EU 12 both in the short and in the long-run under the FMOLS specification.

## 5. Discussion and Policy Implications

These results turn evident the sensitivity of the EKC hypothesis test to the choice of the environmental degradation measure, to the model specification used to test it, and to countries/regions analyzed confirming previous findings [40,41,42,48]. Our findings point that after reaching the turning point of the relationship between economic growth and environmental degradation, in the EU 12, the higher the economic growth of these 12 countries the lower would be the volume of emissions of nitrous oxide, per capita and per area, favoring advances in growth. This happens as well for EU 12, but now only in the short-run and under a different specification, DOLS, this time with methanol emissions decreasing with economic growth after the turning point.

From the results presented in the previous section, we can summarize our findings as follows. Previously, it should be mentioned that a U-shaped relationship between pollutant gases and GDPpc, by area, exists if the coefficient associated with growth is negative and the one associated with economic growth squared is positive, both statistically significant. By opposition, an inverted U-shaped relationship, allowing the validation of the EKC hypothesis, is evident if both statistically significant, the coefficient value of GDPpc/area is positive and the one of GDPpc/area squared is negative.

A U-shaped relationship in the short run is revealed in equation 1 (GHG/area as a dependent; Table 5), under the DFE and FMOLS model for the EU 27, under DFE for the EU 15 and the EU 12, and under the DOLS model solely for the EU 12. In the long run, it seems to be verified solely under DFE and for the EU 15. Considering carbon emissions as a dependent variable (Table 6) in the short run the DFE model results justify the U-shaped relationship for all country groups (EU 27, 15, and 12) and under DOLS this is also validated for EU 12. In the long run only in EU 15 this curve behavior is verified. Turning attention to local pollutants CH_4_ and N_2_O (Table 7 and Table 8, respectively), in the short-run DFE results point for the U-shaped relationship as well in the EU 27 and the EU 12. As well, FMOLS confirms these findings for EU 27. However, in the short-run DOLS results for the EU 12 and the FMOLS results in the long-run validate the EKC hypothesis. Finally, in the short-run, Table 8 presents evidence for the existence of a U-shaped relationship under the DFE model for the EU 27 and the EU 15, whereas for the latter this result is also confirmed under the FMOLS specification. In the long-run, this same U-shaped relationship between N_2_O and economic growth is confirmed for the DFE specification in the EU 12 group. Nonetheless, the inverted U-shaped relationship between environmental degradation and economic growth is confirmed for both the short and the long-run, using the FMOLS specification and only for the EU 12 group.

Thus, we may confirm or validate the EKC hypothesis when local pollutants are used but only in the EU 12 group, not being true when the measure adopted is general GHG emissions nor even carbon emissions, for any of the groups of countries analyzed, independently if we are exploring the results in the short-run or the long-run. The question that seems evident to pose at the moment is why we may observe these differences, or how can we fight the increase in emissions in Europe to fulfill the agreements signed throughout the years. We try to provide some reasoning and suggestions in the following, to understand what else is necessary, based on the existent literature. This raises concerns especially in Europe since we are talking of developed countries, and due to the strong evidence of the U-shaped relationship presented in this article, which contradicts most of the literature analyzing European countries, meaning that a lot more remains to be done at this regard, and as observed results are different depending on the methodology, the geography and the years of entrance in the EU 27 group.

By ratifying the Paris Agreement, the European Union committed to reduce 40% of its GHG emissions by 2030. Furthermore, the European Commission (EC) developed a plan to achieve an EU economy that would be climate neutral in 30 years [86,87]. However, in 2021 there is still a lot remaining to be done in this sense. It is recommended the introduction of new policies combining tools of environmental economics with those of ecological economy using green technologies [88]. Further integration of economic incentives with regulatory changes [88,89], to encourage firms to produce and individuals to consume differently and raising awareness. Only then we could favor our choices of products and services less harmful to the environment to effectively implement environmental policies it is demanded higher economic and financial efforts for countries and their national stakeholders. In sum, concerted efforts at all levels are mandatory, from international, national, and regional organizations, governments, and public authorities, to companies (financial and non-financial), non-governmental institutions, individuals, and households [90]. If further efforts are still needed in the EU 27, in developing countries it is even more urgent [91] and necessary.

In the literature, it has been reported that the EKC does not apply, usually justified by the use of different pollutants as representatives of environmental degradation [92]. Therefore, while some pollution indicators decline over time when economic growth advances, others persistently increase with it. Thus, findings sensitivities are usually reported. Reference [48] explored in depth the empirical literature that has emerged about the EKC. The motivation for keeping digging in the issue is attributed to the mixed results which have been found (different econometric specifications, periods analyzed, countries heterogeneous and specific factors considered, as well as environmental indicators used). It is suggested that policymakers should not encourage continued and unlimited economic growth considering this has not been able to heal environmental problems that countries still have to face. Business activities are just one possible explanation for the EKC hypothesis and shape results. Individuals should be educated in such a way to effectively contribute to environmental protection, especially considering CO_2_ emissions and the results presented.

Using local pollutants is more justifiable due to their internalization in an economy, paving the way for environmental policies able to combat negative externalities. This is harder using global measures since the externalization of problems globally takes a longer period for national policies to correct. Thus, if we need to verify the correct internalization of policies and study if the EKC hypothesis is verified, we need to consider local emission variables or local pollutants representatives and not just global ways of measurement like CO_2_ or global GHG emissions. Our results under the two latter measures, which are widely used in previous empirical studies, show that European countries are still not able to produce more and grow, without seriously harming the environment. Reference [93] tested the EKC validity considering seven emissions indicators and they do not validate it for any of these in China. For a deeper review of articles employing the EKC, we as well suggest the reading of the recent work of [23] who alert to the fact that more research on EKC aligned with green and sustainable technology science is required. A good allowances market function in the EU case will be strictly necessary, but the structure for reducing the level of emissions will depend on technological progress, changes in the sectoral composition, and innovations to boost the technical effect on the production of goods and simultaneously being capable of reducing the abatement costs [9]. Not just increasing production will be the solution, provided that learning by doing innovation processes can create opportunities for balanced growth, controlling, on the one hand, environmental quality and, on the other, providing knowledge for technological development, as supported by [9]. Higher carbon taxes, carbon capture, and further enhancement of the emissions trading scheme are still necessary.

Even if the nature of air pollution is changing, with household air pollution declining since 1990, mostly due to the substitution of energy fossil fuel sources by renewable sources [3,25], the offsetting of these gains in developed countries like the European countries, is driven by the rapid expansion of megacities, industrial production globalization, pesticide and toxic chemicals proliferation, and to the growing use of motor vehicles [2,7]. These all have harmful effects on the health of individuals due to the extensive pollution they still carry [21,22,24]. Additionally, ambient air pollution is responsible for great economic losses. These include higher medical expenditures and the loss of economic productivity, a result of pollution-driven diseases and premature deaths. Pollution is also responsible for the high cost of environmental degradation, even if these costs are largely invisible [2], being spread across large populations over many years [7,8,9]. They even destroy natural resources, several times taken for granted, and are not duly considered in the economic growth process as they should. We believe this is what is happening in the EU given the above-exposed results. Therefore, the inverted U-shaped curve detected only for the EU 12 group might be a disguised illusion of the development stage undergone by these countries. Testing for cubic effects would highlight this since the achievement of the rules imposed to adhere to the EU can be responsible for what the results have shown. These costs combined are so large that they can distort the health system spending and sabotage the growth prospect of countries, which seems to be happening already in the EU 15 given the presented results.

Still, ambient air pollution is not an unavoidable consequence of modern economic growth [27,32,35,56,65,67,74,88,91], but the global requirement of eliminating the ambient air pollution will require courageous and fearless leadership, considerable new resources, mainly financial, from the EU community, and sweeping societal changes such as education and awareness [23,40,48,52,57]. Financial development, industrialization, the industrial sector, and urbanization are pointed in the literature as drivers of CO_2_ emissions increases [23], and only renewable energy was found to reduce the environmental burden up to this moment in an effective way, at least for developed countries such as the EU 27 (see [23] for a comprehensive review of findings). Renewable energy is still pointed out as a solution to reduce GHG emissions in the EU countries, and to end up the still reliance on fossil fuel markets [53,63].

Our results favor the EKC relationship but only in the most recently added EU countries (EU 12). They are at their early stages of development and to continuing being part of the EU group they seem to be taking advantage of the already implemented measures throughout Europe. However, no cubic relationship between economic growth and pollution has been included under the current settings and this could be one of the possible explanations for the results obtained. This informs policymakers in the EU of the emergency in defining policies adapted at the current development stage of the country and not just imposing general ones, unable to simultaneously ensure economic growth and reduce the environmental burden as evidenced in our results. Above we have suggested valuable measures but each proposal should be done at the country or region level and not for the overall EU 27. Moreover, policies should be redirected first for local pollutants, but higher efforts are needed for global gas emissions reduction. Concerning, CO_2_, the emissions trading scheme should be enhanced and spread to the entire EU 27 region, if the goal is to reach the environmental desired targets, while still allowing for economic growth. New technologies to achieve carbon neutrality in terms of 100% of the energy produced by renewable energy sources have to be developed further, and only then in the long term, the global warming threat would stop worrying policymakers and by alleviating the environmental burden, will ensure appropriate population health and wellbeing. To sum up, stricter policy measures and higher demands for the adoption of the best environmental practices in the EU 27 are required to generate an inverted U-shaped curve relationship between GDP and environmental degradation.

## 6. Conclusions

This paper explores the relationship between economic degradation and economic group for the set of the EU 27 countries. Besides, we explore individually the impact of different representatives of pollution and environmental degradation, resorting to four different proxies (GHG, CO_2_, CH_4_, and N_2_O). Both short and long-run effects are explored through DFE, DOLS, and FMOLS techniques during the period 2008–2018, and considering besides the complete set of the EU 27 and the older forming countries, EU 15, and the newly entered EU 12, individually. A profound discussion of results is presented considering previous empirical findings and based on the presented results.

The relatively high GDP of some of the EU 27 countries leads to environmental improvements in those countries but only when CH_4_ and N_2_O emissions were considered in the analysis. From the results obtained we may validate the EKC hypothesis when local pollutants are used but only in the EU 12 group, not being true when the measure adopted are general GHG emissions or even carbon emissions, for any of the groups of countries analyzed, be it in the short-run or the long-run. Independently of the new strategies to be followed or policies to be implemented, they should be supported through subsidies and tax credits. Regarding our CO_2_ findings, further developments in the emissions trading schemes should be as well developed or even transformed maybe expanding it mandatorily for other economic activity sectors. Worldwide and regarding all literature findings provided up to this moment, an effective reduction of environmental degradation, ensuring the fulfillment policies regarding emissions decreases, will only be possible if we can sweep away from fossil-fuel energy consumption. This demands for increased technological development, constant innovation, further financial resources, individuals and businessman, education and awareness, that up to now still seem to be lacking, or not being correctly implemented and administered.

There is still the need to apply longer time series for individual, country groups, or regional analyses to test for the EKC, provided it is still the best way to explore the link between environmental pollution and economic growth. In the policy framework, local pollutants seem to be a better choice to analyze the effectiveness of national policies being implemented. Stimulation of unlimited economic growth is also not a solution to effectively fight environmental issues within both developing and developed countries, as argued by [48]. Using a composite index to represent environmental performance distinguishing between pollutants and emissions would be a reliable extension to the huge amount of empirical evidence that can be found in the literature.

## Figures and Tables

**Table 1:** Variables Synthesis.

Acronym	Variables Selected	Source
GHG pc/area	Greenhouse Gases per capita by surface area (sq. km)	Eurostat
LEdu pc/area	The percentage of the working-age population with an advanced level of education, per capita, and per area	World Bank
GDP pc/area	Gross domestic product per capita (pc) and per area	Eurostat
GDP pe^2^/area	Gross domestic product squared per capita and per area	
Euse/area	Energy use (kg of oil equivalents) per capita and per area	World Bank *
Elect/area	Electricity production from oil, gas, and coal sources (% of total) per capita and per area	World Bank *
CO_2 pc_/area	Volume emissions of carbon per capita and per area	Eurostat/World Bank *
CH_4 pc_/area	Volume emissions of methanol per capita and per area	
N_2_O pc/area	Volume emissions of nitrous oxide per capita and per area	

Notes: * Ratios were computed by the authors using original data from the cited sources. All variables are in the natural log form. The volume of emissions of carbon per capita (CO_2_), emissions of methanol per capita (CH_4_), and emissions of nitrous oxide per capita (N_2_O).

**Table 2:** Cross dependence (CD) test results by European group.

	Cross Dependence Test		
	Europe 27	Europe 15	Europe 12
GHG pc/Area	48.36 ***	27.78 ***	22.64 ***
CO_2_ pc/Area	47.23 ***	26.75 ***	19.73 ***
CH_4_ pc/Area	46.28 ***	23.92 ***	21.34 ***
N_2_O pc/Area	34.38 ***	17.42 ***	16.30 ***
GDP pc/Area	43.43 ***	21.21 ***	20.92 ***
GDPpc^2/Area	15.37 ***	3.24 ***	11.95 ***
Edu pc/Area	10.27 ***	23.34 ***	−1.24
Euse pc/Area	40.36 ***	22.15 ***	17.20 ***
Elect pc/Area	35.40 ***	26.53 ***	9.09 ***

Notes: *** represents statistically significant at 1%, respectively. The volume of emissions of carbon per capita (CO_2_), emissions of methanol per capita (CH_4_), and emissions of nitrous oxide per capita (N_2_O).

**Table 3:** Unit root test results for level and first differences, first and second-generation, by a panel of countries: EU 27, EU 15, and EU 12, with and without a trend.

**Independent Variables**	**Unit Root (First Generation) Panel EU 27 Countries**		**Unit Root (First Generation) Panel EU 15 Countries**		**Unit Root (First Generation) Panel EU 12 Countries**	
**Level**	**Without T**	**With Trend**	**Without T**	**With Trend**	**Without T**	**With Trend**
GHG pc/Area	18.619	57.391	11.626	31.145	6.993	26.246
CO_2_ pc/Area	27.061	62.435	18.225	41.148 **	8.806	21.237
CH4 pc/Area	6.198	304.885 ***	2.613	163.794 ***	3.585	141.091 ***
N_2_O pc/Area	34.175	108.859 ***	18.768	39.911 **	15.407	68.948 ***
GDP pc/Area	7.16	353.888 ***	6.194	187.396 ***	0.966	166.492 ***
GDPpc^2/Area	9.338	285.478 ***	7.37	124.259 ***	1.967	161.218 ***
Edu pc/Area	65.694	45.328	25.932	18.954	39.763 ***	26.774
Euse pc/Area	42.878	67.337 **	18.648	42.211 **	24.23	25.126 **
Elect pc/Area	53.353	44.12	21.634	25.518 **	31.72	18.602
**1st Difference**						
GHG pc/Area	48.508	103.455 ***	39.335	37.261	9.174	66.194 ***
CO_2_ pc/Area	45.173	104.912 ***	32.934	50.328 **	12.239	54.584 **
CH4 pc/Area	168.220 ***	164.549 ***	154.304 ***	70.429 ***	13.916	94.120 ***
N_2_O pc/Area	130.955 ***	172.838 ***	122.939 ***	147.36 ***	8.016	25.474 **
GDP pc/Area	108.255 ***	215.946 ***	104.53 ***	128.54 ***	3.667	87.405 ***
GDPpc^2/Area	126.258 ***	301.138 ***	116.719 ***	158.052 ***	9.539	143.086 ***
Edu pc/Area	50.097	73.506 **	34.202	23.155	16.077	50.351 ***
Euse pc/Area	66.048	80.186 **	24.21	36.954	41.838 **	43.231 ***
Elect pc/Area	149.773 ***	134.525 ***	21.07	23.135	128.702 ***	111.386 ***
	**CIPS (2nd Generation)** **Panel EU 27 Countries**		**CIPS (2nd Generation)** **Panel EU 15 Countries**		**CIPS (2nd Generation)** **Panel EU 12 Countries**	
**Level**	**Without T**	**With Trend**	**Without T**	**With Trend**	**Without T**	**With Trend**
GHG pc/Area	−1.413 **	1.779	−1.678 **	0.216	−1.536 **	1.029
CO_2_ pc/Area	−1.710 **	1.255	−1.087	0.136	−0.789	1.234
CH4 pc/Area	1.32	2.578	1.858	2.337	0.403	0.987
N_2_O pc/Area	−5.372 ***	−3.277 ***	−3.435 ***	−0.547	−4.787 ***	−4.099 ***
GDP pc/Area	1.527	2.042	2.424	2.1	−0.118	0.533
GDPpc^2/Area	1.22	4.757	3.318	4.358	0.487	2.713
Edu pc/Area	0.8	1.9229	0.367	1.693	0.556	0.671
Euse pc/Area	−4.983 ***	−1.857 ***	−4.135 ***	−1.761 **	−3.042 ***	−0.995
Elect pc/Area	−6.187 ***	−3.502 ***	−6.431 ***	−4.170 ***	−2.401 ***	−0.37
**1st Difference**						
GHG pc/Area	−0.851	4.223	−0.562	1.87	−2.344 ***	1.984
CO_2_ pc/Area	−1.923 **	2.82	−2.098 **	0.721	−2.213 **	1.172
CH4 pc/Area	0.057	2.361	−18.59 **	1.743	−3.282 **	−5.008 ***
N_2_O pc/Area	−2.476 ***	−2.515 ***	0.229	0.361	−3.427 ***	−3.941 ***
GDP pc/Area	−2.633 ***	1.601	−3.476 ***	1.125	−2.008 **	1.055
GDPpc^2/Area	−3.075 ***	1.932	−1.470 **	2.209	−0.758 **	0.33
Edu pc/Area	1.219	2.28	1.739 **	2.52	2.448	−0.603
Euse pc/Area	−3.954 ***	−3.380 ***	−2.905 ***	−0.579	−4.619 ***	−2.985 ***
Elect pc/Area	−7.805 ***	−7.364 ***	−4.964 ***	−4.760 ***	−5.060 ***	−4.565 ***

Notes: **, *** represents statistically significant at 5% and 1%, respectively. The volume of emissions of carbon per capita (CO_2_), emissions of methanol per capita (CH_4_), and emissions of nitrous oxide per capita (N_2_O). T stands for trend.

**Table 4:** Pedroni’s and Kao cointegration test results.

**Pedroni’s Test**	**Panel EU 27 Countries**		**Panel EU 15 Countries**		**Panel EU 12 Countries**	
	**Equation (1)**	**Equation (2)**	**Equation (1)**	**Equation (2)**	**Equation (1)**	**Equation (2)**
Mod. Phillips Perron t	6.6527 ***	6.6938 ***	5.2964 ***	5.3117 ***	4.4489 ***	4.4807 ***
Phillips Perron tt	−7.9001 ***	−7.1108 ***	−8.1219 ***	−8.1236 ***	−5.4209 ***	−4.3485 ***
Aug Phillips Perron t	−6.3086 ***	−5.7236 ***	−4.5552 ***	−4.4756 ***	−4.3701 ***	−3.5815 ***
	**Equation (3)**	**Equation (4)**	**Equation (3)**	**Equation (4)**	**Equation (3)**	**Equation (4)**
Mod. Phillips Perron t	7.1765 ***	−7.0877 ***	5.5036 ***	5.1923 ***	4.9666 ***	4.8758 ***
Phillips Perron tt	−10.6922 ***	−15.709 ***	−7.0111 ***	−10.943 ***	−9.1800 ***	−13.4892 ***
Aug Phillips Perron t	−6.1829 ***	−8.1480 ***	−3.8430 ***	−6.2365 ***	−4.9779 ***	−5.2494 ***
**Kao Test**	**Panel EU 27 Countries**		**Panel EU 15 Countries**		**Panel EU 12 Countries**	
	**Equation (1)**	**Equation (2)**	**Equation (1)**	**Equation (2)**	**Equation (1)**	**Equation (2)**
Mod.Dickey Fuller t	1.0005	1.0379	0.1485	−0.5267	0.1432	0.6231
Dickey Fuller t	0.039	0.0043	−1.5926 **	−2.5184 ***	−0.3511	0.2918
Aug Dickey Fuller t	2.1531 **	2.229 **	0.7408	0.7337	1.3666 *	1.5287 *
	**Equation (3)**	**Equation (4)**	**Equation (3)**	**Equation (4)**	**Equation (3)**	**Equation (4)**
Mod.Dickey Fuller t	2.1318 **	−1.9748 **	0.9705	0.2954	1.2293 *	−2.9982 ***
Dickey Fuller t	1.5853 **	−4.3673 ***	−0.1616	−1.8037 **	0.9906	−3.8259 ***
Aug Dickey Fullert	1.7641 **	−4.8482 ***	0.6989	−2.0112 **	0.6286	−4.0194 ***

Notes: *, **, *** represents statistically significant at 10%, 5% and 1%, respectively.

**Table 5:** DFE, DOLS, and FMOLS results for the total sample (EU-27), subsample 1 (EU-15), subsample 2 (EU-12) regarding Equation (1).

Dependent: GHG	Total Sample (EU 27)			Subsample 1 (EU 15)			Subsample 2 (EU 12)		
Independent	DFE	DOLS	FMOLS	DFE	DOLS	FMOLS	DFE	DOLS	FMOLS
D.L. GDPpc	−0.3759 ***	−0.8405 ***	−0.3916 ***	−0.6195 ***	−1.0230 ***	−0.6624 ***	−0.1382 **	−0.7980 ***	−0.0999
D.L. GDPpc^2^	0.0368 **	−0.0189	0.0149	0.0324 **	−0.0493	−0.0197	0.0761 ***	0.1119 **	0.0609 ***
D.L. LEdu	0.4068	2.0318 ***	0.4590 ***	0.9178 ***	2.1424 ***	0.8466 ***	0.2332	2.7779 ***	0.1624 **
D.L. Euse	0.4948 ***	0.3379 **	0.4845 ***	0.2749 ***	0.3379 **	0.2546 ***	0.6552 ***	0.2453 **	0.6207 ***
D.L. Elect	0.0842 ***	0.0264 **	0.0849 ***	0.0854 ***	0.1075 ***	0.1214 ***	0.1016 ***	0.2301 **	0.0945 ***
Constant	4.0172 *	0.0269	0.0077	12.0509 **	0.1309	0.0490	7.4067 *	0.0825	0.0936
ECT	−0.4432 ***			−0.4221 ***			−0.5145 ***		
L. GDPpc (−1)	−1.1079 ***	−0.0049	−0.0079 ***	−0.7840 ***	0.0012	0.0013	−1.0529 ***	−0.0238	−0.0132 **
L. GDPpc^2^ (−1)	−0.0351 **	−0.0003	0.0006	0.0864 **	0.0015 *	0.0003	−0.0366 **	−0.0043 *	−0.0037 ***
L. Ledu (−1)	1.2019 ***	0.0024	0.0121	2.3253 ***	−0.0053	0.0130	1.1755 **	0.0289	0.0016
L. Euse (−1)	0.3031 **	0.0063	−0.0089	0.1113 **	−0.0103	−0.0265 ***	0.5058 ***	−0.0546	−0.0438 *
L. Elect (−1)	0.1347 ***	0.0052	0.0022	0.0501	−0.0073	−0.0087 **	0.1875 **	0.0071	0.0135
Observations		270	269		150	149		120	119
R^2^		0.7353	0.4745		0.8379	0.5359		0.9095	0.3881

Notes: *, **, *** mean that values are statistically significant at 10%, 5% and 1%, respectively. D.L. - Differenced Lagged; —Lagged; ECT—Error Correction Term; Dynamic Fixed Effects (DFE); Dynamic Ordinary Least Squares (DOLS); Fully Modified Ordinary Least Squares (FMOLS). The rest of the variables’ acronyms were defined in Table 1. Estimations of Equation (1).

**Table 6:** DFE, DOLS, and FMOLS results for the total sample (EU-27), subsample 1 (EU-15), subsample 2 (EU-12) regarding Equation (2).

Dependent: CO_2_	Total Sample (EU 27)			Subsample 1 (EU 15)			Subsample 2 (EU 12)		
Independent	DFE	DOLS	FMOLS	DFE	DOLS	FMOLS	DFE	DOLS	FMOLS
D.L. GDPpc	−0.2722 ***	−0.8432 ***	−0.3015 ***	−0.5160 ***	−1.1708 ***	−0.6125 ***	−0.0200 **	−0.7878 **	0.0327
D.L. GDPpc^2^	0.0436 **	−0.0372	0.0161	0.0920 **	−0.1100	−0.0290	0.0868 ***	0.1597 **	0.0738 ***
D.L. LEdu	0.3882	2.0778 ***	0.5068 ***	0.9066 **	1.9985 ***	0.8712 ***	0.3375	2.8010 ***	0.3174
D.L. Euse	0.5923 ***	0.3989 *	0.5699 ***	0.3293 ***	−0.1574	0.3035 ***	0.7705 ***	0.1608	0.7327 ***
D.L. Elect	0.1051 ***	0.0218	0.1164 ***	0.1156 ***	0.1291	0.1583 ***	0.1263 ***	−0.1654	0.1300 ***
Constant	4.9117	0.0014	−0.0128	15.6942 **	0.0932	0.0932	9.9218 *	0.0744	0.0260
ECT	−0.4253 ***			−0.4670 ***			−0.4481 ***		
L. GDPpc (−1)	−1.1001 ***	−0.0007	−0.0059	−0.6800 ***	0.0008	0.0008	−1.1173 ***	−0.0338 *	−0.0126
L. GDPpc^2^ (−1)	−0.0362 *	−0.0001	−0.0006	0.0909 **	0.0013	0.0013	−0.0388 *	−0.0057 **	−0.0047 ***
L. Ledu (−1)	1.2289 ***	0.0092	0.0111	2.4549 ***	0.0187	0.0187	1.4484 **	0.0498 *	0.0150
L. Euse (−1)	0.3734 **	0.0864	−0.0073	0.1546	−0.0194	−0.0194	0.5591 **	−0.0818 *	−0.0066 **
L. Elect (−1)	0.1521 ***	0.0036	0.0016	0.0845	−0.0068	−0.0068	0.2280 **	0.0071	0.0140 **
Observations		270	269		150	149		120	119
R^2^		0.6991	0.3946		0.7998	0.4197		0.8968	0.3344

Notes: *, **, *** mean that values are statistically significant at 10%, 5% and 1%, respectively. D.L.—Differenced Lagged; L—Lagged; ECT—Error Correction Term; Dynamic Fixed Effects (DFE); Dynamic Ordinary Least Squares (DOLS); Fully Modified Ordinary Least Squares (FMOLS). The rest of the variables’ acronyms were defined in Table 1. Estimations of Equation (2).

**Table 7:** DFE, DOLS, and FMOLS results for the total sample (EU-27), subsample 1 (EU-15), subsample 2 (EU-12) regarding Equation (3).

Dependent: CH_4_	Total Sample (EU 27)			Subsample 1 (EU 15)			Subsample 2 (EU 12)		
Independent	DFE	DOLS	FMOLS	DFE	DOLS	FMOLS	DFE	DOLS	FMOLS
D.L. GDPpc	−0.8881 ***	−0.9936 ***	−0.8172 ***	−0.9583 ***	−0.8943 ***	−0.8508 ***	−0.8881 ***	1.2386 ***	−0.7725 ***
D.L. GDPpc^2^	0.0170 **	0.0122	0.0166 *	−0.0004	−0.0029	0.0211	0.0170 **	−0.0312 **	0.0201
D.L. LEdu	−0.4560	0.8758 **	0.1231	0.5373 ***	0.3043	0.6988 ***	−0.4560	2.3745 ***	−0.4090 **
D.L. Euse	0.1572 **	0.2553 **	0.1204 ***	−0.0144	0.3353	0.0629	0.1572 **	−0.3453	0.1820 ***
D.L. Elect	0.0567 **	0.0457	0.0267 **	0.0080	−0.0395	0.0036	0.0567 **	−0.0436	0.0568 ***
Constant	−9.1797 **	0.0584	0.0801 **	−1.0121	0.3786 ***	0.2480 ***	−9.1797	−0.5936 *	0.2453
ECT	−0.2945 ***			−0.1101 ***			−0.2945 ***		
L. GDPpc (−1)	−1.5085 ***	−0.0022	−0.0139 ***	−1.5118 ***	0.0152 *	0.0032	−1.5085 ***	−0.0125	0.0246 ***
L. GDPpc^2^ (−1)	−0.0674 **	−0.0006	−0.0007 **	−0.0108	0.0018 **	0.0008	−0.0674 **	−0.0024 **	−0.0094 **
L. Ledu (−1)	0.2964	0.0183 *	0.0208 **	1.9607 **	−0.0610 **	−0.0257 *	0.2964	0.1118 ***	0.0271
L. Euse (−1)	−0.2487	−0.0253 ***	−0.0198 ***	−0.8382	0.0136	−0.0019	−0.2487	−0.0724 ***	−0.0252 *
L. Elect (−1)	0.0772	0.0002	0.0047 **	0.1435	−0.0004	−0.0023	0.0772	−0.0080	0.0073
Observations		270	269		150	149		120	119
R^2^		0.8480	0.7763		0.9386	0.8634		0.9034	0.7567

Notes: *, **, *** mean that values are statistically significant at 10%, 5% and 1%, respectively. D.L.—Differenced Lagged; —Lagged; ECT—Error Correction Term; Dynamic Fixed Effects (DFE); Dynamic Ordinary Least Squares (DOLS); Fully Modified Ordinary Least Squares (FMOLS). The rest of the variables’ acronyms were defined in Table 1. Estimations of Equation (3).

**Table 8:** DFE, DOLS, and FMOLS results for the total sample (EU-27), subsample 1 (EU-15), subsample 2 (EU-12) regarding Equation (4).

Dependent: N_2_O	Total Sample (EU 27)			Subsample 1 (EU 15)			Subsample 2 (EU 12)		
Independent	DFE	DOLS	FMOLS	DFE	DOLS	FMOLS	DFE	DOLS	FMOLS
D.L. GDPpc	−0.4484 ***	−0.7971 ***	−0.5732 ***	−0.5189 **	−1.1725 **	−0.4877 ***	−0.2825	−0.9424 *	0.1910 **
D.L. GDPpc^2^	0.0791 **	0.0070	0.0560	0.1011 **	−0.0345	0.0994 **	0.0887 **	0.1085	−0.0101 **
D.L. LEdu	0.2400	1.9833 **	0.4336	1.7643	4.5508 ***	1.7466 ***	−0.5439	4.5412 ***	0.5945
D.L. Euse	−0.2060 **	0.5140 *	−0.2797 ***	−0.0066	−1.0901 *	−0.1214	−0.3595 ***	0.5059	−0.3873 ***
D.L. Elect	0.0069	−0.4192 ***	−0.0412	−0.0356	0.0190	−0.0269	−0.0298	−0.4630 **	−0.0402
Constant	1.6176	0.1849 *	0.0895	18.6430 **	0.6457 ***	0.0106	−0.3428	−0.1676	0.0940
ECT	−0.6707 ***			−0.7228 ***			−0.6468 ***		
L. GDPpc (−1)	−1.3264 ***	0.0044	−0.0127	−0.9670 ***	−0.0131	−0.0043	−1.3908 ***	−0.0233	0.1175 ***
L. GDPpc^2^ (−1)	−0.1041 ***	−0.0001	−0.0016	0.0237	0.0029 *	0.0014	0.1261 ***	−0.0028	−0.0052 ***
L. Ledu (−1)	1.8511 ***	−0.0027	−0.0070	2.9738 ***	−0.0023	0.0150	1.6360 **	0.0182	0.0786
L. Euse (−1)	−0.0470	−0.0305	−0.0015	−0.0981	−0.0051	−0.0238	0.0263	−0.0042	0.0107
L. Elect (−1)	−0.0332	0.0039	0.0032	−0.1214*	0.0027	−0.0033	−0.0206	0.0063	0.0301 **
Observations		270	269		150	149		120	119
R^2^		0.6077	0.2015		0.7391	0.2354		0.8963	0.3121

Notes: *, **, *** mean that values are statistically significant at 10%, 5% and 1%, respectively. D.L.—Differenced Lagged; —Lagged; ECT—Error Correction Term; Dynamic Fixed Effects (DFE); Dynamic Ordinary Least Squares (DOLS); Fully Modified Ordinary Least Squares (FMOLS). The rest of the variables’ acronyms were defined in Table 1. Estimations of Equation (4).

## Data Availability

Data has been collected from publicly archived datasets analyzed or generated during the study and presented in Table 1.
